# EP Receptor Expression in Human Intestinal Epithelium and Localization Relative to the Stem Cell Zone of the Crypts

**DOI:** 10.1371/journal.pone.0026816

**Published:** 2011-10-25

**Authors:** Lene Th. Olsen Hult, Charlotte R. Kleiveland, Kjetil Fosnes, Morten Jacobsen, Tor Lea

**Affiliations:** 1 Molecular Cell Biology Group, Department of Chemistry, Biotechnology and Food Science, Norwegian University of Life Sciences, Aas, Norway; 2 Ostfold Hospital, Fredrikstad, Norway; German Institute for Human Nutrition, Germany

## Abstract

There is substantial evidence for PGE2 affecting intestinal epithelial proliferation. PGE2 is also reported to be involved in the regulation of growth and differentiation in adult stem cells, both effects mediated by binding to EP-receptors. We have used the Lgr5 as a marker to scrutinize EP-receptor and COX expression in human intestinal epithelial cells with focus on the stem cell area of the crypts. Normal tissue from ileum and colon, but also duodenal biopsies from patients with untreated celiac disease, were investigated by immunohistochemistry and RT-PCR. The combination of fresh flash-frozen tissue and laser microdissection made it possible to isolate RNA from the epithelial cell layer, only. In the small intestine, Lgr5 labels cells are in the +4 position, while in the colon, Lgr5 positive cells are localized to the crypt bottoms. Epithelial crypt cells of normal small intestine expressed neither EP-receptor mRNA nor COX1/2. However, crypt cells in tissue from patients with untreated celiac disease expressed EP2/4 receptor and COX1 mRNA. In the colon, the situation was different. Epithelial crypt cells from normal colon were found to express EP2/4 receptor and COX1/2 transcripts. Thus, there are distinct differences between normal human small intestine and colon with regard to expression of EP2/4 receptors and COX1/2. In normal colon tissue, PGE2-mediated signaling through EP-receptors 2/4 could be involved in regulation of growth and differentiation of the epithelium, while the lack of EP-receptor expression in the small intestinal tissue exclude the possibility of a direct effect of PGE2 on the crypt epithelial cells.

## Introduction

There is growing evidence that prostaglandins, and PGE2 in particular, affect intestinal epithelial cell proliferation and apoptosis [1]. The two cyclooxygenase (COX) isoforms, COX-1 and COX-2, both catalyze the conversion of arachidonic acid (AA) into the intermediates PGG2 and PGH2, that subsequently acts as substrate for specific prostaglandin (PG) synthases and the formation of the different prostanoids [2]. COX-1 is suggested to be constitutively expressed in most cells and tissues under normal circumstances. COX-2 is usually absent or only weakly expressed, but is induced in response to inflammatory mediators, growth factors, oncogene activation and tumor promoters [3]. PGE2 is the most prominent prostaglandin and mediates its effects by binding to E prostanoid receptors (EP receptors). Four different EP receptors exist, EP1-4, which are all G-protein coupled receptors (GPCRs).

Deficiency of endogenous prostaglandins due to inhibition of the COX enzymes by nonsteroidal anti-inflammatory drugs (NSAIDs) is important for ulcerogenic response in the intestine [4]. It has been reported in mouse models that PGE2 stimulates intestinal epithelial growth [4], [5]. Recent studies in mouse models of DSS colitis suggest that preservation of epithelial proliferation depends on PGE2 production [6], [7]. PGE2 is also found to exert growth-stimulatory effects on intestinal tumors, and exogenous administration of PGE2 provides a growth advantage to intestinal neoplasms [8], [9]. On the other hand, disruption of the COX-2 or the E-prostanoid receptor 2 (EP2) gene result in a substantial reduction of polyps in APC knockout mice [10], [11].

The absorptive epithelium of the small intestine is organized in crypts and villi, and is the most rapidly self-renewing tissue in adult mammals. Villous epithelium contains three types of mature epithelial cells; the enterocytes, the goblet cells and the enteroendocrine cells. The crypts are mainly occupied by undifferentiated rapidly proliferating cells in addition to Paneth cells and a functional stem cell compartment [12], [13].

PGE2 has been reported to be important for the regulation of growth and differentiation in haematopoietic and mesenchymal stem cells [14], [15]. Furthermore, recent data suggest PGE2 to be part of a master switch for growth regulation in somatic stem cells, in general [16]. Wnt signalling which has been established as the major signalling pathway driving proliferation in the intestinal epithelium [17], is affected by PGE2 trough a PKA dependent pathway leading to GSK3β inhibition and β-catenin stabilization [15], [16]. EP2 and 4 are known to be coupled to Gs and thereby elicit elevation of cAMP and PKA activation in the cell [18]. By this mechanism PGE2 is able to contribute to Wnt driven proliferation. Effect of PGE2 on proliferation depends on EP receptor expression and the presence of PGE2 producing cells.

Detailed transcriptional studies of pure human epithelial cells have been hampered by the inability to properly dissect the tissue. Most molecular insight concerning EP receptor and COX expression has been performed in whole mucosal tissue, which includes a variety of cells. Some successful experiments combining laser microdissection with immunohistochemistry on thin sections have been reported, but RNA qualities have been poor [19]. In this study we combined fresh flash-frozen tissue and cryosectioning at −20° with a novel, gentle post-fixation and staining protocol before laser microdissection. A slightly modified commercial RNA isolation method resulted in RNA of acceptable quality for cDNA synthesis and RT-PCR from as few as 200 cells.

The suggested role of PGE2 in regulation of growth and differentiation of adult stem cells and the reports of PGE2 as a stimulator of intestinal epithelial growth, led us to investigate the possibility of PGE2 affecting human intestinal stem cells. In this work we have identified COX1/2 and EP-receptor expressing cells and investigated how these are localized relative to Lgr5 positive putative stem cells in human small intestine and colon tissue. Epithelial EP receptor and COX expression patterns were identified in normal and inflamed human small intestine and normal large intestine.

## Methods

### Immunohistochemistry

Tissue was immersed in 4% formalin for 2–3 hours within 10–20 minutes after removal from the patient. Then the tissue was dehydrated in a graded ethanol series and infiltrated in paraffin. Tissue sections (7 µm) were obtained with a Leica RM2245 rotary microtome (Leica Microsystems, Germany) and mounted on coated glass slides. Sections were then de-waxed in Histolene clearing agent for 3×5 minutes at room temperature (RT). Tissue sections were rehydrated through a graded ethanol series before antigen retrieval. Antigen retrieval were performed for 20 minutes in boiling 1M sodium citrate buffer pH 6.0, followed by cooling to RT for 20 minutes. For the anti-EP and CD45 immunohistochemistry experiments antigen retrieval was performed using IHC-Tek Epitope Retrieval Solution (IHC World, Woodstock, MD) and an IHC-Tek Epitope Retrieval Steamer set (IHC World, Woodstock, MD). Slides were incubated in the steamer for 40 minutes followed by 20 minutes at RT. Remaining buffer was gently wiped off before the tissue specimens were encircled with a PAP pen and placed in a humid chamber. The tissue sections were incubated for 1 hour at RT with blocking solution, 5–7% of secondary antibody host serum in TBS-T buffer, before primary antibody solution was added. Incubation with primary antibody was performed overnight at 4°C. Primary antibody solution was removed and sections were washed briefly in TBS-T buffer before secondary antibody solution was added. Sections were incubated with secondary antibody for 1 hour at RT in the dark. Sections were again washed in TBS-T buffer, before the nuclear stain Hoechst 3342 was added for 10 minutes at RT (Sigma-Aldrich, St Louis, MO). Slides were mounted either with immersion oil or ProLong Gold antifade reagent (Invitrogen, UK).

The following primary and secondary antibodies were used in this study;

Rabbit polyclonal anti- GPR49/LGR5 (#LS-A1232 and #LS-A1236, MBL, Woburne, MA), goat polycolonal anti-COX-1 and goat polyclonal anti-Cox-2 (M19)[20] (Santa Cruz Biotechnology, Santa Cruz, CA), mouse monoclonal CD133 (clone AC133) (Miltenyi Biotech, Germany)[21], donkey anti-rabbit IgG FITC, rabbit anti-goat IgG Texas Red, goat anti-mouse IgG Cy3, goat anti-rabbit IgG FITC (Jackson ImmunoResearch, UK). Rabbit polyclonal anti-human Prostaglandin E2 receptor EP1 subtype, rabbit polyclonal anti-human Prostaglandin E2 receptor EP2 subtype, rabbit polyclonal anti-human Prostaglandin E2 receptor EP3 subtype, rabbit polyclonal anti-human Prostaglandin E2 receptor EP4 subtype (Genway, CA), monoclonal mouse CD45 (clone 2B11) (DAKO, Denmark)[22], [23], mouse KI67 antigen (clone MM1)[24] (Monosan, Netherlands), goat anti-mouse IgG-FITC (Southern Biotech, Birmingham, AL). All slides were examined with a Leica SP5 confocal laser microscope (Leica Microsystems, Germany). Antibody blocking peptides used in the study were; PTGER1 petide (GWB-TK4210) PTGER2 peptide (GWB-4S87FM), PTGER3 peptide (GWB-J273CX), GWB-38WU1H (Genway, San Diego, CA) and Cox2 (M19)P (Santa Cruz Biotechnology, Santa Cruz, CA).

### Tissue/Biopsies

The collection of tissue samples have been approved by Regional Committees for Medical and Health Research Ethics (REK). All patients have given their written consent on consent form approved by the committee. Normal ileal tissue was obtained from the terminal ileum of patients undergoing right hemicolectomy resections for colon cancer, and normal colon was obtained from sigmoidal resections in patients with rectopexia. Inflamed small intestinal tissue was dissected from patients with untreated coeliac disease.

### Laser microdissection (LMD)

Tissues from human colon and small intestine were collected during scheduled operations at the clinic, oriented, and flash-frozen in liquid nitrogen. Tissue samples were mounted in Tissue-Tek mounting media (Sakura, Torrance, CA) in a cryostat at −20°C. 8 µm sections were obtained at −20°C, and collected on PEN (polyethylene naphthalate) membrane slides for microdissection (Leica Microsystems, Germany). Sections were briefly dried at RT, before post-fixed in ice-cold acetone for 2 minutes and washed in ice-cold diethylene pyrocarbonate (DEPC) treated dH_2_O. Sections were stained with Toluidine Blue in DEPC-treated dH_2_O for 1 minute and washed. Sections were left to dry at RT for approximately 5 minutes. Excess liquid was gently wiped off.

Laser micro dissection were performed on a Leica LMD 7000 (Leica Microsystems, Germany), and all removable parts were wiped off with RNAse®Zap solution prior to dissection (Ambion Applied Biosystems, Austin, TX). RNAse free 0.5 ml vials were mounted in the collection device.

**Figure 1:**
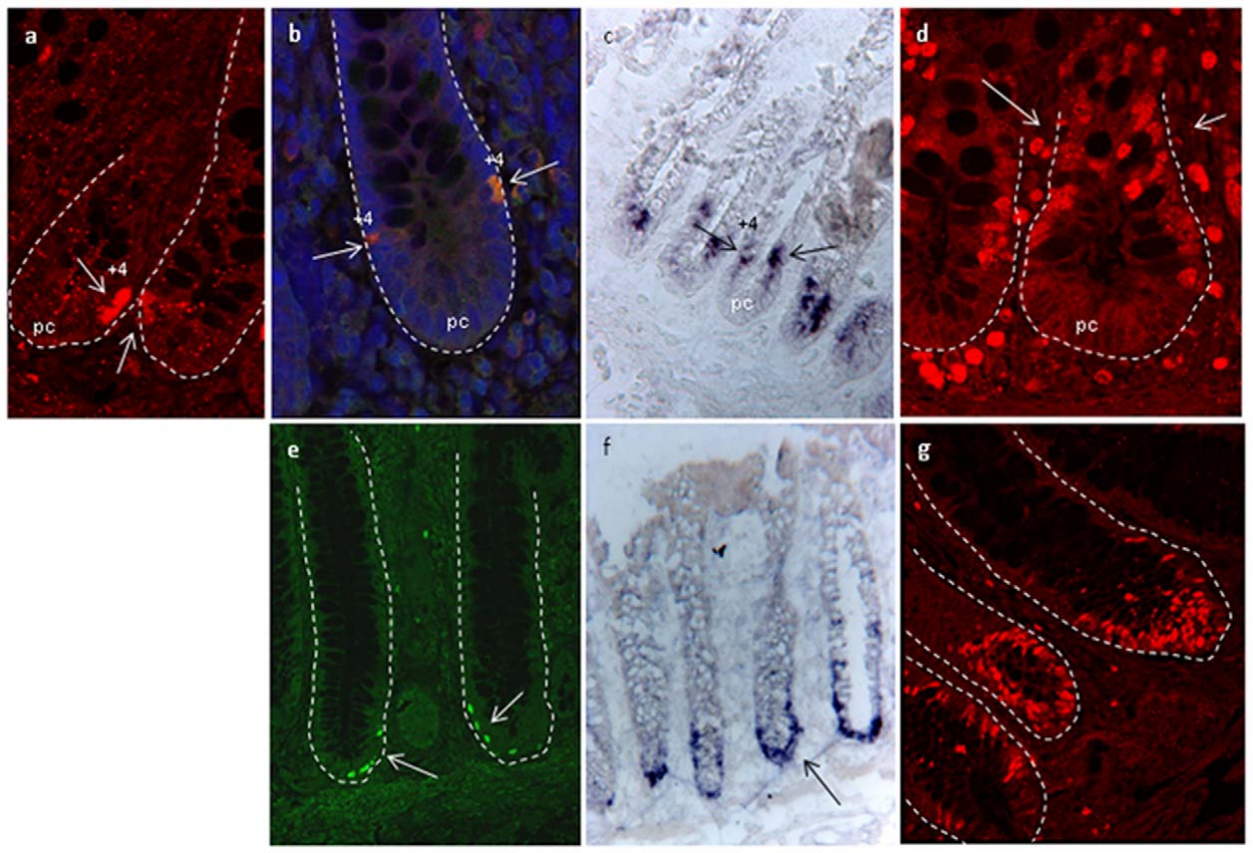
Identification of putative stem cells. Paraffin sections of normal human small intestinal tissue were prepared for immunohistochemical analysis with **a)** anti-CD133 antibody identifies several positive cells from +4 position and upwards (indicated by arrows), **b)** anti-Lgr5 antibody identify positive cells in +4 positions (indicated by arrows). The nuclei were stained with Hoechst 33342, **c)** In situ hybridization of normal human small intestine for Olfm4 reveals an expression pattern similar to the expression of Lgr5, in the +4 position, **d)** Sections of normal human small intestinal tissue were prepared for immunohistochemical analysis with anti-Ki67 antibody to identify proliferating cells. Positive cells are indicated with arrows (pa  =  Paneth cells), **e)** Paraffin sections of normal human colon tissue were prepared for immunohistochemical analysis with anti-Lgr5 antibody, and positive cells are identified in the crypt base (indicated by arrows), **f)** In situ hybridization of normal human colon for Olfm4 reveals an expression pattern similar to the expression of Lgr5 at the crypt base. **g)** Sections of normal human colon tissue were prepared for immunohistochemical analysis with anti-Ki67 antibody to identify proliferating cells.

Normal intestinal tissue crypts were divided into two populations based on localization, the *crypt high* population was dissected from position +6 and upwards while the *crypt low* population was dissected from position +5 and downwards and include the crypt base (Figure 1A). Ten different templates were utilized in these experiments; normal small intestinal crypt high, normal small intestinal crypt low, normal small intestine below +4, inflamed small intestine crypt cells, inflamed total tissue, small intestine total tissue, normal colon crypt high, normal colon crypt low and normal colon total tissue. Total tissue in this experiment means a mixture of crypt epithelial cells and lamina propria cells. Cells belonging to the different populations were dissected out according to manufacturer's recommendations and collected in tubes containing RLT lysis buffer (Qiagen, Germany), and incubated on ice for approximately 1 hour.

### RNA extraction and cDNA synthesis

Microdissected cells were collected in 65 µl RLT lysis buffer with mercaptoethanol according to manufacturer's recommendations. RNA was extracted according to the manual for Qiagen RNeasy Micro Kit (Qiagen, Germany), LMD tissue. RNA was eluted in 14 µl RNAse-free water. cDNA synthesis was performed by using SuperScript® VILO™ cDNA Synthesis Kit (Invitrogen, UK) according to the manufacturer's recommendations. cDNA synthesis program: 25°C 10 minutes, 42°C 90 minutes, 85°C 5 minutes, 4°C forever (MJ Thermocycler, Watertown, MA).

### RT-PCR

PCR was performed using SYBR GreenER qPCR reaction system (Invitrogen, UK) according to the manufacturer's recommendation. Template cDNA was always measured on a Nanodrop to be 300 ng (Thermo Scientific, Waltham, MA). All PCR reactions were analyzed using a Rotor Gene 6000 Real-Time PCR Machine (Corbett Life Science). The following PCR program was used:

50°C 2 minutes, 95°C 5 minutes, 95°C 15 seconds, 60°C 1 minute (40 cycles of two last steps). Melting curve analyses were performed for all real-time PCR runs to verify amplification of only one product and to exclude false positive results.

The following primers were used in this study:

EP1: Qiagen QuantiTect Primer Assay, Hs_PTGER1_1_SG

EP2_forward: GTGCTGACAAGGCACTTCAT

EP2_reverse: GTCACTGTTTGGGGTTTCAA

EP3_forward: GGATCATGTGTGTGCTGTCC

EP3_reverse: AACTGGAGACAGCGTTTGC

EP4_forward: GACCTGTTGGGCACTTTGTT

EP4_reverse: TGGACGCATAGACTGCAAAG

Lgr5_forward: CTCTTCCTCAAACCGTCTGC

Lgr5_reverse: GATCGGAGGCTAAGCAACTG

Cox1_forward: GAGTACTGGAAGCCGAGCAC

Cox1_reverse: GCACTCTGGAATGACAAGCA

Cox2_forward: CTAGAGCCCTTCCTCCTGTG

Cox2_reverse: AAAACTGATGCGTGAAGTGC

DEFA5_forward: GCCATCCTTGCTGCCATT

DEFA5_reverse: GCTTCTGGGTTGTAGCCTCATC

GAPDH_forward: GAGTCAACGGATTTGGTCGT

GAPDH_reverse: TTGATTTTGGAGGGATCTCG

RPII_forward: GCACCACGTCCAATGACAT

RPII_reverse: GTGCGGCTGCTTCCATAA

### In situ hybridization

cRNA probes were generated from Olfm4 cDNA from normal human small intestine using RNA labelling kit (SP6/T7) according to the manufacturer's protocol (Roche Applied Science, Indianapolis, IN). 12 µm cryo sections from normal human small intestine or colon were post fixed with 4% paraformaldehyde for 20 min at RT. The sections were then treated with 0.1% active DEPC in PBS for 2×15 min before 15 min equilibration in 5×SCC pH 7.5. Prehybridization was continued for 2 h at 63°C before a 24 h hybridization step with 500 ng/ml probe at 63°C. The sections were then washed 30 min at room temperature with 2×SCC, 1 h with 2×SCC at 65°C and 1 h with 0.1×SCC at 65°C. Equilibration in Tris/NaCl buffer with 0.1% Tween 20 was done before 2 h incubation with anti-DIG antibody (1∶2000) (Roche Applied Science) in blocking buffer (Roche Applied Science). Washing was done in Tris/NaCl buffer for 2×15 min before 5 min equilibration in NTM buffer. Incubation with substrate was done over night in the dark. The slides were then mounted and investigated by light microscopy.

## Results

### Identification of putative stem cells in the crypts of human small and large intestine

For PGE2 to affect epithelial cell proliferation the receptors must be expressed together with intestinal stem cells or transit amplifying (TA) cells. The exact location of the intestinal stem cells (ISCs) has remained controversial. This has primarily been due to the lack of unique molecular markers. The glycoprotein CD133 is expressed in immature hematopoietic stem cells, as well as other stem and progenitor cells including neural and embryonic stem cells [25], [26]. Monoclonal antibodies against CD133 have also been used to identify colon cancer stem cells [27], [28]. The expression of CD133 in normal human intestine was investigated by immunohistochemistry. In normal small intestine, we found CD133 positive cells among the epithelial crypt cells, as well as in cells adjacent to the crypts. The CD133 positive cells detected in the crypts were found in the lower parts of the crypts, i.e. in the vicinity of the suggested intestinal stem cell positions (Fig. 1a). No CD133 positive cells were detected in normal colon crypts (data not shown). Controls with secondary antibody, only, were consistently negative (Figure S1a–b).

**Figure 2:**
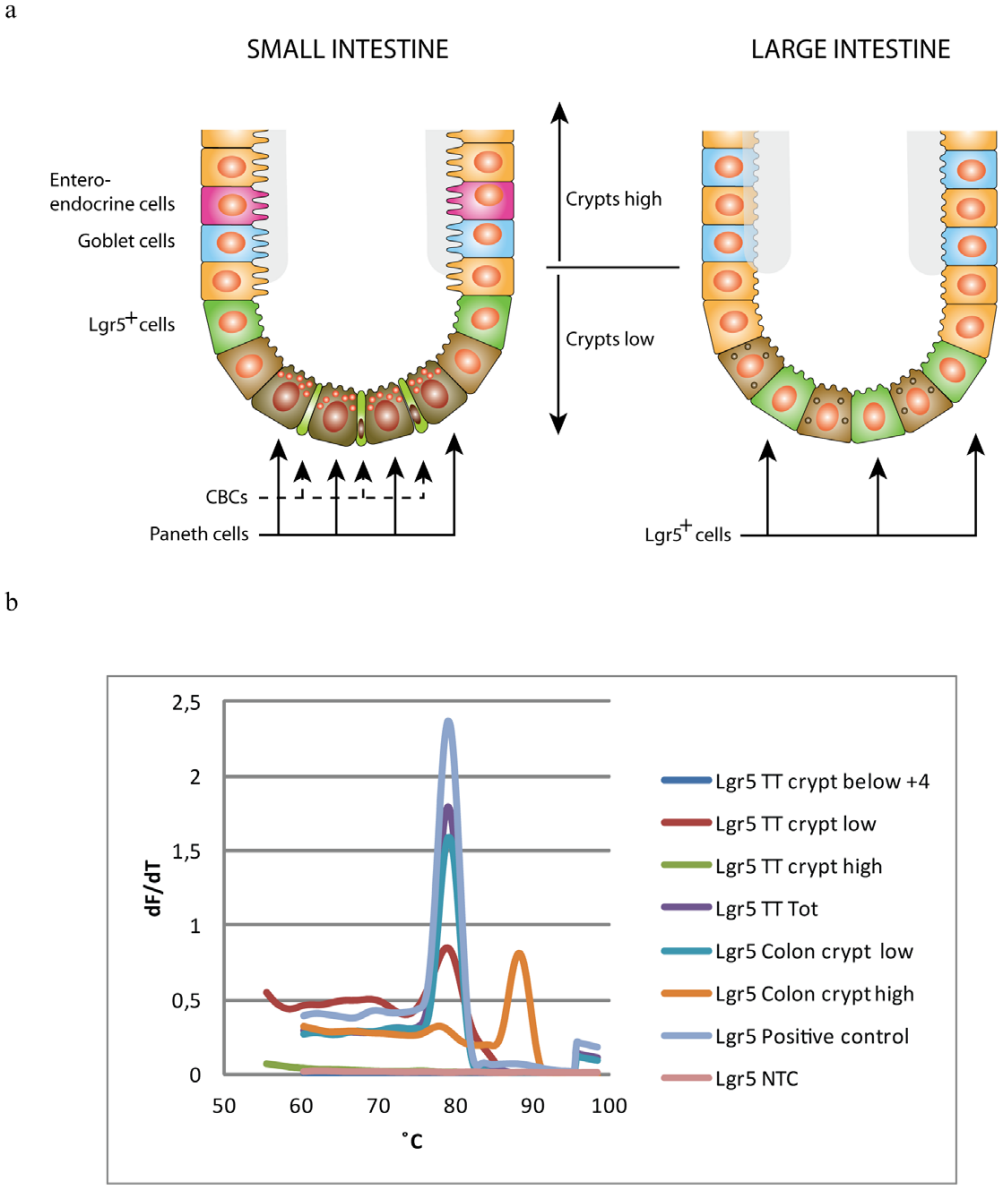
Lgr5 gene expression in normal human intestine. **a)** Schematic illustration of how the crypts of human intestinal tissue were divided into different populations**, b)** Cells from the different populations described above were dissected by LMD from normal human small intestine and colon, and inflamed small intestinal tissue. RNA were isolated from the dissected cells and analyzed by RT-PCR for the expression of Lgr5. Amplification of Lgr5 PCR producs were verified by correct melting temperature.

The orphan G-protein coupled receptor Lgr5/GPR49 has recently been shown to specifically label stem cells in the mouse small intestine as well as other adult tissues [29]. In this study, antibodies against Lgr5/GPR49 were used to localize the predicted intestinal stem cells of the human small and large intestine. By using a combination of two antibodies raised against Lgr5/GPR49, one against the N-terminal extracellular domain (LS-A1232) and one against the C-terminal domain (LS-A1236), we obtained a satisfactory bright and precise positive signal. In crypts of the small intestine we detected Lgr5 positive cells at approximately position +4, directly above the Paneth cells at the crypts base (Fig. 1b). The anti-Lgr5 antibodies failed to detect Lgr5 expression in crypt base columnar (CBC) cells that are localized between the Paneth cells in murine models [29]. We also investigated Lgr5 expression by RT-PCR. The finding of Lgr5 positive putative stem cells in position +4 by immunohistochemeistry led to division of the crypts into two populations; *crypts high* above +5 and *crypts low* below +5 (Fig. 2a). Lgr5 transcripts were found in the *crypt low* population which includes cells in the +4 position. Investigation of the cells below the +4 position that included CBC cells and Paneth cells, only, revealed no detectable Lgr5 mRNA expression (Fig. 2b). As expected, no Lgr5 transcripts were detected in the *crypt high* population by PCR.

In human normal colon crypts we detected Lgr5 positive cells at the crypt base, varying from 3–6 positive cells per crypt (Fig. 1c). PCR confirmed Lgr5 expression only in *crypt low* population of normal colon epithelial cells (Figure 2b). Controls with normal rabbit IgG, secondary antibody, only, were consistently negative (Figure S1 e,f,h,i). Immunhistochemistry on U937 cells shown not to express any transcript for Lgr5 confirmed the specificity of the antibody (Figure S2).

Olfactomedin-4 (Olfm4) is reported to be a robust marker of Lgr5+ cells [30]. We performed *in situ* hybridization experiments on cryo sections of normal human small intestine and colon (Fig. 1c and f). Olfm4 is expressed in the +4 position in normal human small intestine and at the crypt base of normal colon tissue. This is the same expression pattern found for Lgr5 using immunohistochemistry. Controls with sense probes were consistently negative (Figure S1 j–k).

### Identification of proliferating cells in intestinal crypts

Both the transit amplifying cells and the intestinal stem cells are proliferating cells and should be positive for the proliferation marker Ki67 by immunhistochemistry. In normal small intestine Ki67 positive cells were identified from position +4 and upwards to the top of the crypt (Fig. 1d and g). In normal colon Ki67 positive cells were identified all the way from the crypt base to the crypt top. This is in accordance with the Lgr5 and Olfm4 data and confirms that no proliferating cells are found below position +4 in the epithelium of normal small intestine.

### Profiling of EP receptor expression by RT-PCR on microdissected crypt cell populations

**Figure 3:**
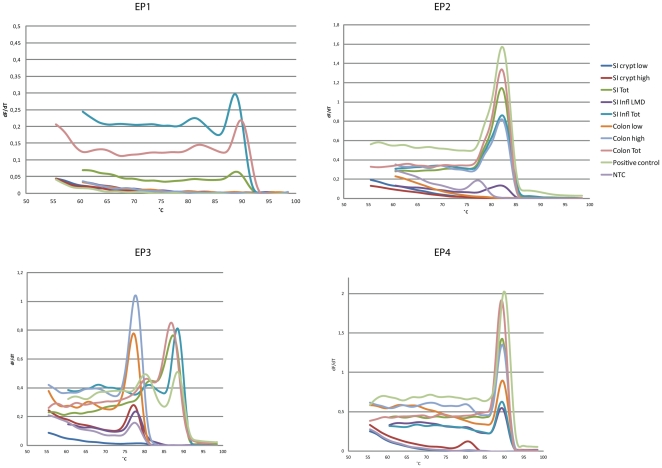
EP-receptor gene expression in normal and inflamed human intestine. Cells from the different populations described above were dissected by LMD from normal human small intestine and colon, and inflamed small intestinal tissue. RNA were isolated from the dissected cells and analyzed by RT-PCR for the expression of EP-receptors 1-4. Amplification of the EP receptor PCR products were verified by correct melting temperature.

Prostaglandins, particularly PGE2, have been shown to be important for stem cell proliferation in vertebrates [14] and to play a role in the regulation of epithelial cell growth in response to injury [6], [7]. PGE2 binds to and mediate signaling through four different receptors, EP1-4. We therefore investigated the EP-receptor mRNA expression profile by RT-PCR. Laser microscopy dissection (LMD) combined with cryosectioning, RNA isolation and cDNA synthesis was followed by RT-PCR. In normal small intestinal crypts we failed to detect transcripts for any of the EP receptors in both the *crypt high* and *crypt low* populations (Fig. 3). When total small intestinal tissue was used as a template, however, all transcripts were expressed. We also investigated inflamed small intestinal tissue from patients with untreated celiac disease (Fig. 3). In epithelial crypt cells from this tissue we were able to detect expression of transcripts for EP-receptors 2 and 4. EP1 and EP3 transcripts were not detected. In the total tissue template from inflamed tissue all transcripts examined were detected.

In epithelial cells from normal colon tissue we detected transcripts of EP2 and EP4 receptors in both *crypt high* and *crypt low* populations. Transcripts of the two reference genes GAPDH and RPII were present in all templates (Fig. 3).

### Identification of EP1–4 and CD45 positive cells in human intestinal tissue

**Figure 4:**
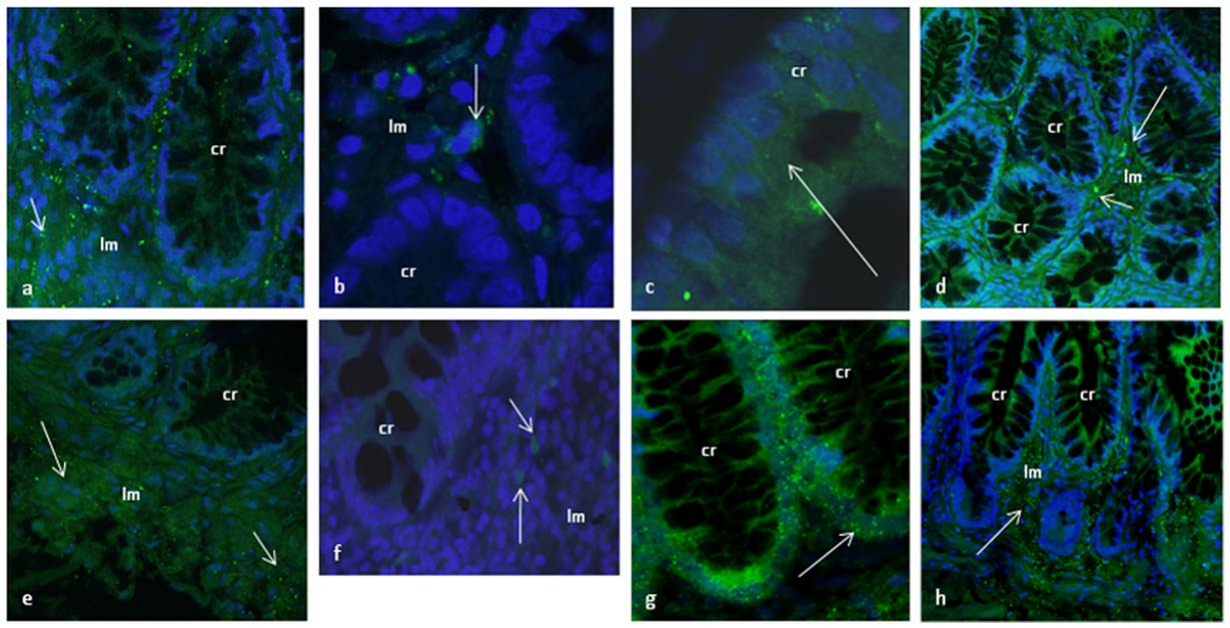
Identification of EP receptors in normal human intestine by immunhistochemistry. **a**–**b)** Anti-EP1 antibody detects positive cells in the lamina propria in both colon (a) and small intestinal tissue. No expression is found in crypts. (b), **c**–**d)** Anti-EP2 antibody detects positive cells in colon crypts (c), but not in small intestine crypts where EP2 expression is detected in lamina propria only (d), **e**–**f)** Anti-EP3 antibody detects positive cells in the lamina propria in both colon (e) and small intestinal tissue (f). No expression is found in crypts, **g**–**h)** Anti-EP4 antibody detects positive cells in colon crypts (g), but not in small intestine crypts where EP4 expression is detected in lamina propria only. Nuclei are stained with Hoechst 33342.

In these experiments we made use of recently developed rabbit polyclonal antibodies raised against peptides from the extracellular domains of the four different EP receptors. We tested these antibodies by immuncytochemistry on a human mesenchymal stem cell line (hTERT) as a positive control (data not shown). We detected EP1- and EP3-positive cells in the lamina propria of both normal colon and small intestine (Fig. 4 a, b, e, f), but no signal was observed in epithelial crypt cells in either tissues. EP2- and EP4-expressing cells were found both in the lamina propria and among epithelial crypt cells of colon (Fig. 4 c, g), but in small intestinal tissue EP2- and EP4-expression was restricted to cells in the lamina propria (Fig. 4 d, h). Controls with normal rabbit IgG, with secondary antibody and peptide inhibition were all consistently negative (Figure S1 d,e, f,g,h,i and S3a–d).

**Figure 5:**
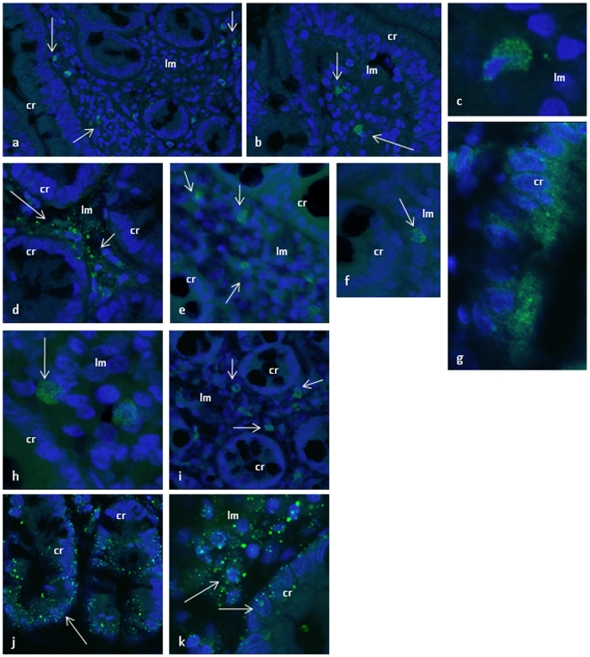
Identification of EP receptors and CD45 in inflamed human small intestine by immunhistochemistry. **a**–**c)** CD45 antibody detected positive CD45 cells in the lamina propria (lm) in inflamed tissue. No positive CD45 cells were detected in crypts (cr), **d**–**e)** Anti-EP1 antibody detected positive cells in the lamina propria, but no EP1 expression were detected in crypt cells, **f**–**g)** Anti-EP2 antibody detected positive cells in both lamina propria (f) and crypts (g), **h**–**i)** Anti-EP3 antibody detected positive cells in lamina propria only; no positive cells were detected in crypts, **j**–**k)** Anti-EP4 antibody detected positive cells in both lamina propria (j) and crypts (k). Nuclei are stained with Hoechst 33342.

The expression of EP-receptors was also investigated in inflamed small intestinal tissue obtained as biopsies from patients with untreated celiac disease. Here the EP1 and EP3 receptor staining pattern of the lamina propria was similar to what was found in normal tissue (Fig. 5 d, e, h, i). However, EP2- and EP4-expression, were clearly detected in epithelial crypt cells of small intestine (Fig. 5 f, g, j,k). To rule out the possibility that the EP2- and EP4-positive crypt cells were intraepithelial lymphocytes we also stained the same tissue with monoclonal anti-CD45 antibodies. Numerous CD45-positive cells were observed in the inflamed lamina propria, but no CD45 positive cells were found in the crypt epithelium (Fig. 5 a–c). Normal mouse and rabbit IgG, secondary antibody and peptid inhibition experiments were performed to ensure antibody specificity (Figure S1 a–b).

### Identification of COX-1 and COX-2 positive cells in human intestinal tissue

**Figure 6:**
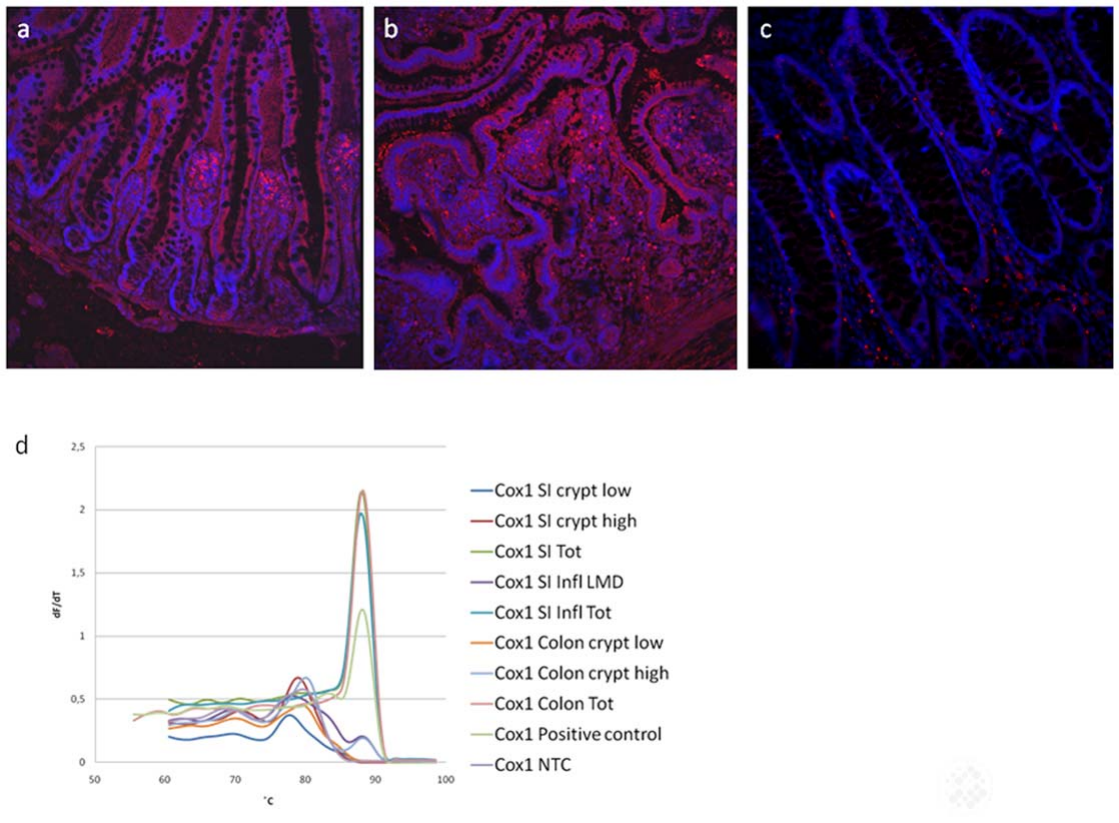
Identification of COX1 in normal and inflamed human intestine. Paraffin sections were prepared for immunohistochemical analysis with anti-COX1 antibodies and the nuclei were stained with Hoechst 33342, **a)** Normal human small intestine showing positive COX1 cells in lamina propria, **b)** Human small intestine from patients with untreated celiac disease showing positive COX1 cells in lamina propria, **c)** Normal human colon showing positive COX1 cells in lamina propria, **d)** Cells from the different populations described above were dissected by LMD from normal human small intestine and colon, and inflamed small intestinal tissue. RNA were isolated from the dissected cells and analyzed by RT-PCR for the expression of COX1. Amplifications of the COX1 PCR products were verified by the correct melting temperature.

PGE2 is produced by the enzymes COX-1/2. COX-1 is regarded as constitutively expressed while COX-2 is induced by different stimuli. We therefore investigated the COX-1 and COX-2 expression and localization in human intestinal tissue. Cellular expression of the COX-1 and COX-2 was investigated by immunohistochemistry in normal and inflamed human small intestinal tissue. We did not detect COX-1 positive cells in the epithelium of normal and inflamed small intestine. A high number of COX-1 positive cells were found in the lamina propria of normal tissue, and the expression was increased in inflamed tissue of untreated celiac disease patients (Fig. 6a and b). COX-1 expression was, however, detected both in epithelial crypt cells and the lamina propria of normal colon (Fig. 6c). These immunohistochemistry data corresponds with mRNA expression data (Fig. 6d).

**Figure 7:**
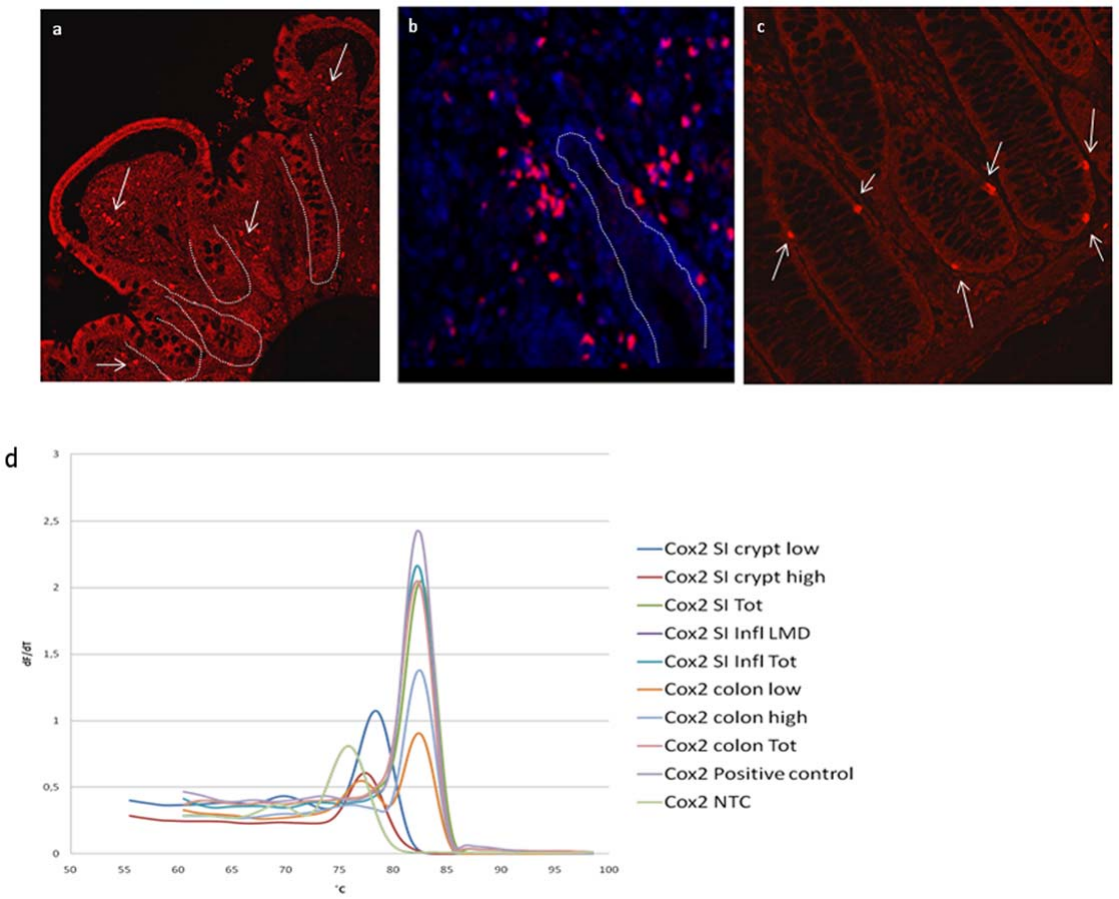
Identification of COX2 in normal and inflamed human intestine. Paraffin sections were prepared for immunohistochemical analysis with anti-COX2 antibody (crypts are indicated with dashed lines). **a)** Normal human small intestine. Positive COX2 cells in the lamina propria are indicated by arrows, **b)** Human small intestine from patients with untreated celiac disease show positive COX2 cells in lamina propria, **c)** Normal human colon. Positive COX2 cells in epithelial crypts are indicated by arrows, **d)** Cells from the different populations described above were dissected by LMD from normal human small intestine and colon, and inflamed small intestinal tissue. RNA were isolated from the dissected cells and analyzed by RT-PCR for the expression of COX2. Amplifications of the COX2 PCR products were verified by correct melting temperature.

In the small intestine a modest number of COX-2 positive cells were detected in or in close proximity to the epithelial cells in the crypts (Fig. 7a). PCR confirmed that these COX2 positive cells were not localized to the epithelium but were lamina propria cells (Fig. 7d). Numerous COX-2 positive cells were detected in the lamina propria of inflamed small intestine (Fig. 7b). As opposed to the situation in the small intestine, COX-2 positive epithelial crypt cells were identified in normal colon (Fig. 7c). Investigation of mRNA expression with qPCR confirmed COX-2 expression in epithelial cells of colon crypts. These cells were randomly scattered among the epithelial crypt cells. No positive cells were detected in the lamina propria of normal colon (Fig. 7d). Peptide blocking experiments were performed to confirm the immunohistochemistry data (Figure S3e-f).

## Discussion

Recent studies in mice suggest that PGE2 is important to maintain mucosal integrity [4], [5], [6], [7], [31]. Inhibition of COX expression in the intestinal mucosa has been found to reduce the levels of PGE2 and inhibit proliferation of intestinal epithelial cells [4]. If PGE2 has a direct effect on intestinal epithelial proliferation this is most likely due to effects on intestinal stem cells and/or transit amplifying cells. The biological effects of PGE2 are mediated through binding to the corresponding EP receptors 1–4. We investigated both the transcript levels of the EP receptors by RT-PCR as well as characterized the distribution and localization of the receptors by immunohistochemistry. Immunhistochemistry and RT-PCR results both conclude that EP2 and EP4 receptors were expressed in colon epithelial crypt cells, whereas EP1 and EP3 receptors were absent. In colon, EP receptors of all the 4 subtypes were expressed on cells of the lamina propria while only EP2- and EP4-expression was detected among the epithelial crypt cells. It is previously reported by Takafuji and co-workers that EP3 receptors is expressed at the apex of normal colonic crypts [32]. However, we are not able to detect EP3 receptor expression in colonic crypts, neither on gene nor protein level.

In normal small intestinal epithelium none of the EP receptors were present, neither at the top nor at the bottom of the crypts. In total mucosal tissue all transcripts were found and all receptors were detected by antibodies. The inflamed epithelium from untreated patients with celiac disease, expressed EP2 and EP4 in crypt cells, but still no EP1 and EP3 expression were observed. However, all receptors were expressed in total mucosal tissue. The transcript distribution found by RT-PCR was fully confirmed by the immunohistochemistry experiments.

The intestinal epithelial layer is known to contain intraepithelial lymphocytes (IEL) and the number of these is expected to increase during inflammation [33]. The detection of EP2 and EP4 receptors in inflamed small intestinal crypts could therefore be due to increased expression of these receptors on IEL. However, staining with antibodies against CD45 did not reveal any positive cells among the epithelial crypt cells. The EP2 and EP4 receptors found in inflamed small intestinal epithelium are therefore assumed to be expressed on epithelial crypt cells.

The EP receptors differ in their affinity for PGE2 and downstream signaling. PGE2 is reported to affect proliferation by interacting with the Wnt signaling pathway trough a PKA dependent inhibition of GSK3β[15], [16]. PGE2 induces elevation of cAMP and subsequent PKA activation by binding to EP2 and/or EP4 receptors. Our findings of EP2 and 4 receptor transcripts in epithelial cells of normal colon tissue and inflamed small intestine make it plausible that PGE2 interacts directly with these cells and modulate their proliferation.

Studies of haematopoietic stem cells show that PGE2 is involved in regulation of the stem cell niche and of the stem cell itself [14]. Also, in human mesenchymal stem cells PGE2 is involved in regulating stem cell proliferation [15]. Until now the exact location of intestinal stem cells has been a controversial issue. This has primarily been due to the lack of unique molecular markers. Recent studies in mouse models have identified a single marker, Lgr5/GPR49, a leucine-rich orphan G-protein-coupled receptor, that specifically labels stem cells in the mouse small intestine as well as other adult tissues [25], [26], [29]. Furthermore, CD133 (Prominin 1) has been used as a colon cancer stem cell marker [34], and it has also been suggested as a reliable marker for intestinal stem cells [35]. In the present study a combination of two different anti-Lgr5 antibodies revealed positive cells in both small intestinal and colon crypts. The localization of these cells is in accordance with previously reported findings of intestinal crypt stem cells in humans [36]. We did not observe co-localization of CD133 positive and Lgr5 positive cells in crypts of the small intestine, and CD133 positive cells were not detected at all in colonic crypts. Studies of Prom1/CD133 knock-in mice have demonstrated Prominin 1 mRNA expression in the lower parts of the crypts, but not overlapping with the Lgr5 transcript expression [37] Our conclusion is that CD133 may be expressed by some putative stem cells, but is not a specific stem cell marker as it also seems to stain early transit amplifying cells and cells in lamina propria. This is in accordance with data from Snippert and collaborators [38].

By immunohistochemistry we identified Lgr5 positive cells in position +4 in human small intestine. We were not able to detect Lgr5 protein expression among the slender cells intercalating between the Paneth cells, also known as the crypt base columnar (CBC) cells, reported by others [29], [39]. This could be explained by Lgr5 being expressed below the detection level of our method, but it could also be due to lack of Lgr5 expression in human CBC cells. Investigation of RNA isolated from cells below the +4 position, including Paneth cells and CBC cells, using LMD and RT-PCR, did not reveal any Lgr5 transcripts. The CBC cells were identified by transmission electron microscopy (data not shown). These two different approaches both suggest that Lgr5 positive cells are only found at position +4 in normal human small intestine. The murine Lgr5-positive CBC cells are reported to be proliferating cells positive for the Ki67 marker [29]. We were not able to detect any Ki67 cells below the +4 position in human small intestine (data not shown). In human colon up to six Lgr5-positive cells were identified at the crypt base. These were either neighbouring cells or separated by 1–2 unidentified cells. Cells intermingling with Lgr5-positive cells in colon are previously suggested to be goblet cells [40]. We primarily observed goblet cells near the top of the crypts, and their frequency decreased gradually towards the crypt base where no positive staining for goblet cells was detected. These cells might be goblet-like or Paneth-like cells, but they do not stain with Dolichos biflorus agglutinin (DBA) or express human defensin alpha 5 (DEFA5) mRNA (data not shown). Evidently, there are differences in how the Lgr5-positive cells are organized in human colon versus small intestine both with regard to the type of cells they are surrounded by, and by number and localization.

In the mouse, crypt stem cell survival has previously been reported to depend on prostaglandins synthesized through COX-1 [41]. Cohn et al. found COX-1 to be constitutively expressed in epithelial cells of normal mouse intestine, and additionally up-regulated in response to injury. However, COX-2 expression has not been reported among the crypt epithelial cells of normal intestine, but is reported to be up-regulated in adenomas and carcinomas as well as in inflammatory bowel disease [42], [43], [44]. In normal human small intestinal tissue we detected a modest number of COX-2 positive cells in the lamina propria close to the crypts. These COX-2 positive cells could be subepithelial myofibroblast-like cells which are expected to be important for regulation of intestinal stem cells [45]. No COX-1 positive cells were identified in normal human small intestine. In normal colon tissue the situation was different. Here, COX-2 positive cells were randomly distributed among the epithelial cells within the crypts, while no positive cells were detected in the surrounding tissue. COX-1 positive cells were found in the lamina propria of normal colon, and also a few crypt cells expressed COX-1. Interestingly, there were more COX-1 and 2 positive cells in the lamina propria of inflamed small intestinal tissue from untreated celiac disease patients. This increase in COX-positive cells could be due to up-regulation of the protein in lamina propria cells or recruitment of COX-positive cells due to the inflammation as reported by [7].

The present study reveals several distinct differences EP receptor-expression of cells in human intestinal epithelium. EP2/4 receptors are all expressed in the epithelium of normal colon crypts, but not in normal small intestinal crypts. These results indicate that PGE2, produced by cells of the intestinal crypt epithelium, may have a direct growth-promoting effect on stem cells and transit amplifying cell of normal human colon. On the other hand, we do not find any evidence for such direct effects on epithelial crypt cells of normal human small intestine. However, we find the levels of these molecules to be up-regulated in inflamed tissue from patients with untreated celiac disease. Thus, inflammation induces expression of these molecules and could represent a mechanism for regulation of growth during inflammatory conditions. Furthermore, there could be indirect growth-regulatory effects of PGE2 via lamina propria cells producing cytokines affecting epithelial crypt cell proliferation and maturation.

We also identified differences in the localization of Lgr5-positive putative stem cells in human colon versus small intestine. Moreover, our data are not in agreement with recent reports from mouse experimental models suggesting that Lgr5-positive stem cells are found at the crypt bottoms of the small intestine.

## Supporting Information

Figure S1 **Control experiments for immunhistochemistry and in situ hybridization experiments.** Paraffin sections were prepared for immunohistochemical analysis with **a.** Rabbit anti-mouse IgG Cy3 secondary antibody only, **b.** inflamed small intestine incubated with normal mouse IgG instead of primary antibody, **c.** inflamed small intestine incubated with goat anti-mouse-FITC secondary antibody only, **d.** inflamed small intestine incubated with normal rabbit IgG instead of primary antibody, **e.** normal small intestine incubated with normal rabbit IgG instead of primary antibody, **f.** normal colon incubated with normal rabbit IgG instead of primary antibody, **g.** inflamed small intestine incubated with goat anti-rabbit-FITC secondary antibody only, **h.** normal colon incubated with goat anti-rabbit-FITC secondary antibody only, **i.** Normal small intestine incubated with goat anti-rabbit-FITC secondary antibody only. All nuclei were stained with Hoechst 33342 **j.** in situ hybridization for Olfm4 normal small intestine, sense control probe, **k.** In situ hybridization for Olfm4 normal colon, sense control probe
(TIF)

Figure S2 **Verification of the specificity of Lgr5 antibody.** Cells were prepared for immunhistochemical analysis with Lgr5 antbody. **a.** Rabbit anti-Lgr5 and goat anti-rabbit-FITC identifies expression of Lgr5 on Caco2 cells. **b.** Rabbit anti-Lgr5 and goat anti-rabbit-FITC identifies expression of Lgr5 on mesenchymal stem cells represented by the hTERT-20 cell line. **c.** No Lgr5 expression could be detected on the U937 cells. Nuclei are stained with Hoechst 3342. **d.** Fragments amplified by PCR were run on an agarose gel; Lgr5 was not amplified from U937 (lane 1), but were detected in both Caco2 and hTERT-20 cells (lane 2 and 3). There was no amplification in the no template control (lane 4). The house keeping transcript RPLPO was amplified in U937, Caco2 and hTERT-20 (lane 5, 6 and 7). NEB 100 bp ladder was used for fragment size determination.
(TIF)

Figure S3 **Control experiments with blocing peptides.** Paraffin sections were prepared for immunohistochemical analysis with **a.** Rabbit anti-EP1 incubated with EP1 peptide which resulted in complete loss of positive EP1 cells in human colon. **b.** Rabbit anti-EP2 incubated with EP2 peptide which resulted in complete loss of positive EP2 cells in human colon. **c.** Rabbit anti-EP3 incubated with EP3 peptide which resulted in complete loss of positive EP3 cells in human colon. **d.** Rabbit anti-EP4 incubated with EP4 peptide which resulted in complete loss of positive EP4 cells in human colon. **e.** Goat anti-COX2 incubated with COX2 peptide resulted in complete loss of positive COX2 cells in human normal colon. **f.** Goat anti-COX2 incubated with COX2 peptide resulted in complete loss of positive COX2 cells in human small intestine. The corresponding peptide was used with a 1000x molar excess in all experiments.
(TIF)
