# Supplementary figures and images for: EP Receptor Expression in Human Intestinal Epithelium and Localization Relative to the Stem Cell Zone of the Crypts
Source: PLoS One. 2011 Oct 25;6(10):e26816. doi: 10.1371/journal.pone.0026816 (PMC3201980; doi:10.1371/journal.pone.0026816)

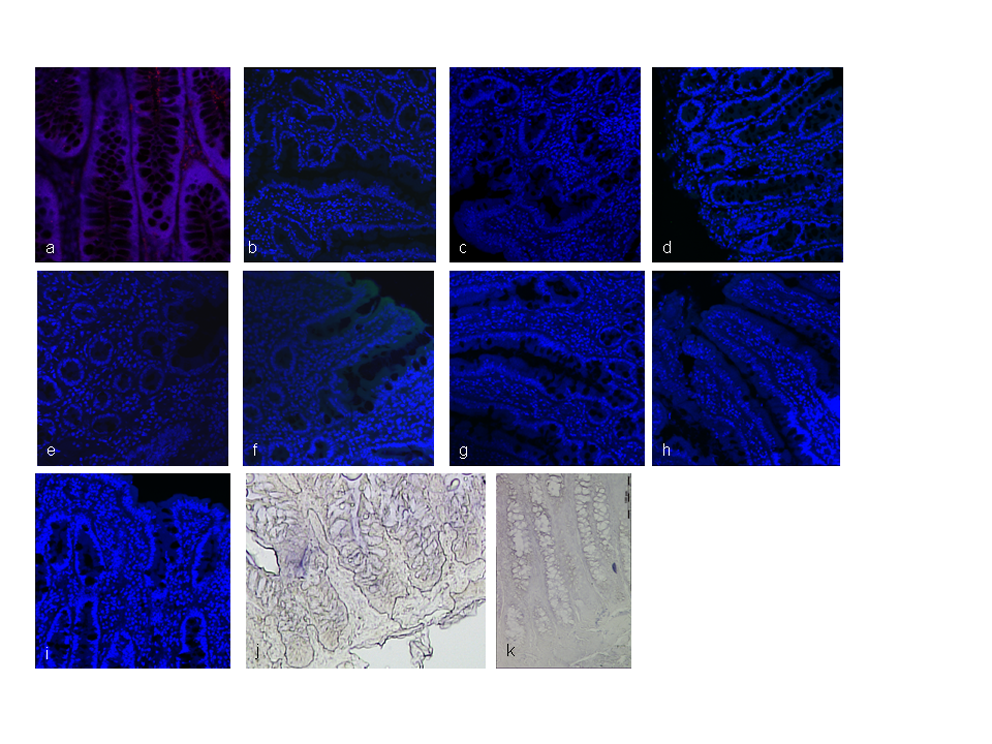

Supplement: Figure S1 — Control experiments for immunhistochemistry and in situ hybridization experiments. Paraffin sections were prepared for immunohistochemical analysis with a. Rabbit anti-mouse IgG Cy3 secondary antibody only, b. inflamed small intestine incubated with normal mouse IgG instead of primary antibody, c. inflamed small intestine incubated with goat anti-mouse-FITC secondary antibody only, d. inflamed small intestine incubated with normal rabbit IgG instead of primary antibody, e. normal small intestine incubated with normal rabbit IgG instead of primary antibody, f. normal colon incubated with normal rabbit IgG instead of primary antibody, g. inflamed small intestine incubated with goat anti-rabbit-FITC secondary antibody only, h. normal colon incubated with goat anti-rabbit-FITC secondary antibody only, i. Normal small intestine incubated with goat anti-rabbit-FITC secondary antibody only. All nuclei were stained with Hoechst 33342 j. in situ hybridization for Olfm4 normal small intestine, sense control probe, k. In situ hybridization for Olfm4 normal colon, sense control probe (TIF) [file pone.0026816.s001.tif]

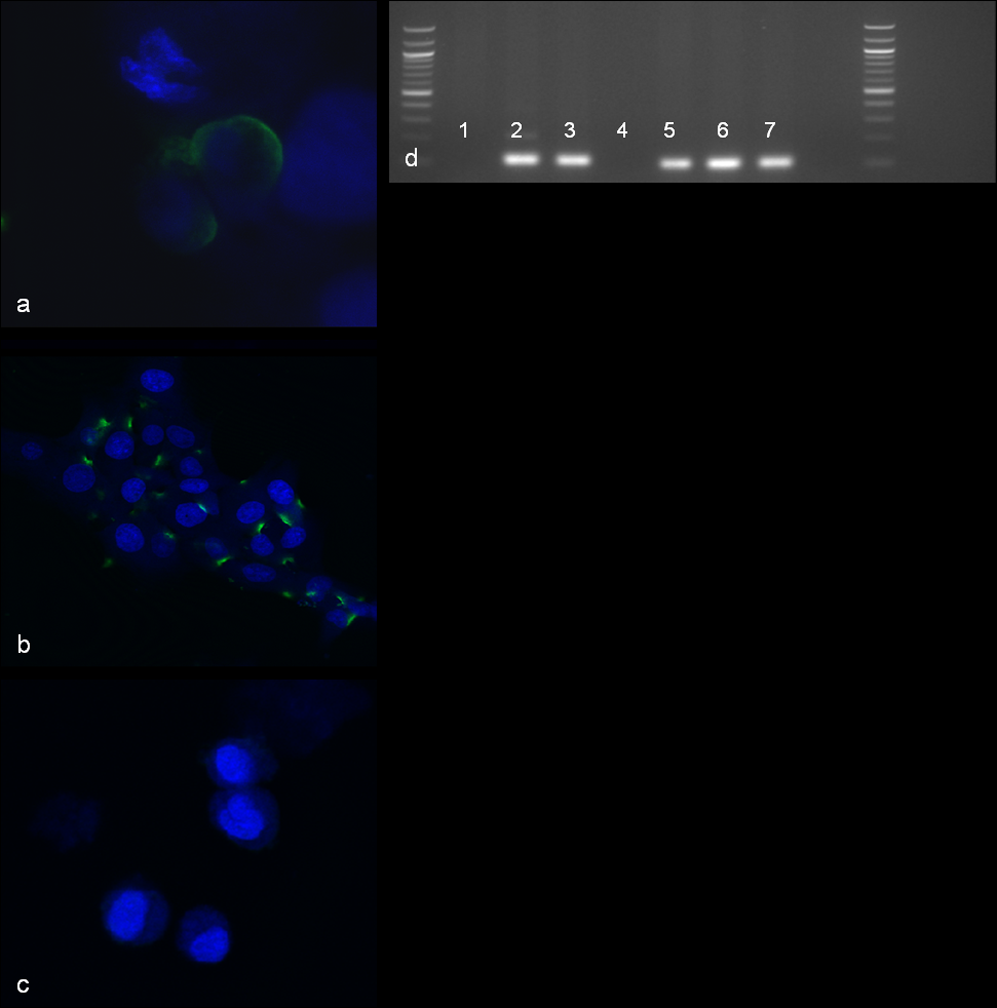

Supplement: Figure S2 — Verification of the specificity of Lgr5 antibody. Cells were prepared for immunhistochemical analysis with Lgr5 antbody. a. Rabbit anti-Lgr5 and goat anti-rabbit-FITC identifies expression of Lgr5 on Caco2 cells. b. Rabbit anti-Lgr5 and goat anti-rabbit-FITC identifies expression of Lgr5 on mesenchymal stem cells represented by the hTERT-20 cell line. c. No Lgr5 expression could be detected on the U937 cells. Nuclei are stained with Hoechst 3342. d. Fragments amplified by PCR were run on an agarose gel; Lgr5 was not amplified from U937 (lane 1), but were detected in both Caco2 and hTERT-20 cells (lane 2 and 3). There was no amplification in the no template control (lane 4). The house keeping transcript RPLPO was amplified in U937, Caco2 and hTERT-20 (lane 5, 6 and 7). NEB 100 bp ladder was used for fragment size determination. (TIF) [file pone.0026816.s002.tif]

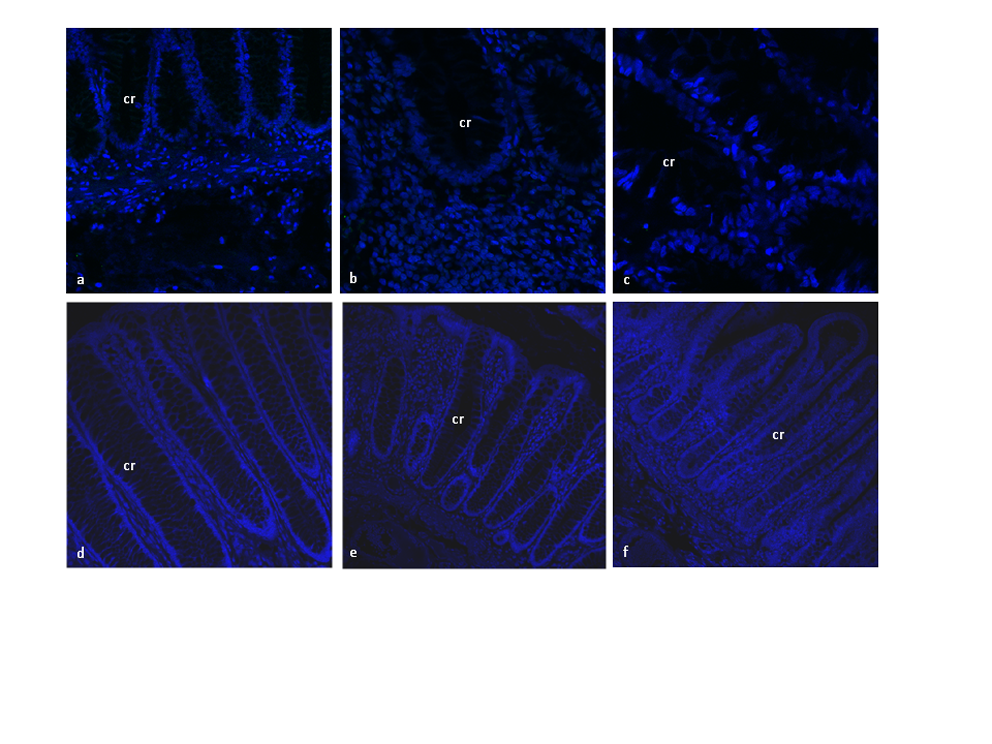

Supplement: Figure S3 — Control experiments with blocing peptides. Paraffin sections were prepared for immunohistochemical analysis with a. Rabbit anti-EP1 incubated with EP1 peptide which resulted in complete loss of positive EP1 cells in human colon. b. Rabbit anti-EP2 incubated with EP2 peptide which resulted in complete loss of positive EP2 cells in human colon. c. Rabbit anti-EP3 incubated with EP3 peptide which resulted in complete loss of positive EP3 cells in human colon. d. Rabbit anti-EP4 incubated with EP4 peptide which resulted in complete loss of positive EP4 cells in human colon. e. Goat anti-COX2 incubated with COX2 peptide resulted in complete loss of positive COX2 cells in human normal colon. f. Goat anti-COX2 incubated with COX2 peptide resulted in complete loss of positive COX2 cells in human small intestine. The corresponding peptide was used with a 1000x molar excess in all experiments. (TIF) [file pone.0026816.s003.tif]
